# Advances in Hydrogel Tissue Engineering for Spinal Cord Injury Repair

**DOI:** 10.1002/smmd.70034

**Published:** 2026-04-30

**Authors:** Ruixing Shui, Fan Ding, Dapeng Li, Guoqing Pan

**Affiliations:** ^1^ Department of Spine Surgery Affiliated Hospital of Jiangsu University Zhenjiang Jiangsu China; ^2^ School of Materials Science and Engineering Institute for Advanced Materials, Jiangsu University Zhenjiang Jiangsu China

**Keywords:** hydrogel, spinal cord injury, tissue engineering, treatment of spinal cord injury

## Abstract

Spinal cord injury (SCI), which is a severe complication of spinal fractures, often causes the dysfunction of the spinal cord and results in sensory and motor abnormalities. Current clinical treatments—including medication, decompression surgery, and bed rest—remain insufficient for complete functional recovery. It is necessary to reduce the early inflammatory reactions, rebuild the connections of neurons, and reduce the formation of the glial scar in order to restore spinal cord function. With the development of biomaterials discipline, hydrogel tissue engineering has become an effective and feasible method. Injectable and highly biocompatible hydrogel can directly fill the injured site as a scaffold material that can provide physical support to reduce scar formation and promote axon growth. In addition, hydrogels have the ability to regulate pathophysiological events. For example, it can reduce inflammatory reactions, inhibit glial scar formation, and promote axonal growth, so as to achieve the recovery of motor function after SCI. This review systematically correlates the four pathological phases of SCI with the stage‐specific biological functions of hydrogels. It summarizes the current state of research in SCI and hydrogel‐based tissue engineering, and discusses the key challenges and future directions in this evolving field.

## Highlights

- This review introduces the pathophysiology of various stages of SCI and current clinical treatments for SCI.
- This review focuses on the applications of hydrogels in SCI treatment and their molecular biological roles during different stages of SCI.
- This review summarizes the current situation of hydrogel in SCI treatment, and prospects its future development direction.

## Graphical abstract

This review systematically introduces the pathological process of spinal cord injury (SCI) and the applications and biological roles of hydrogels in SCI repair. Emerging 4D printing and gene therapy strategies are also discussed.

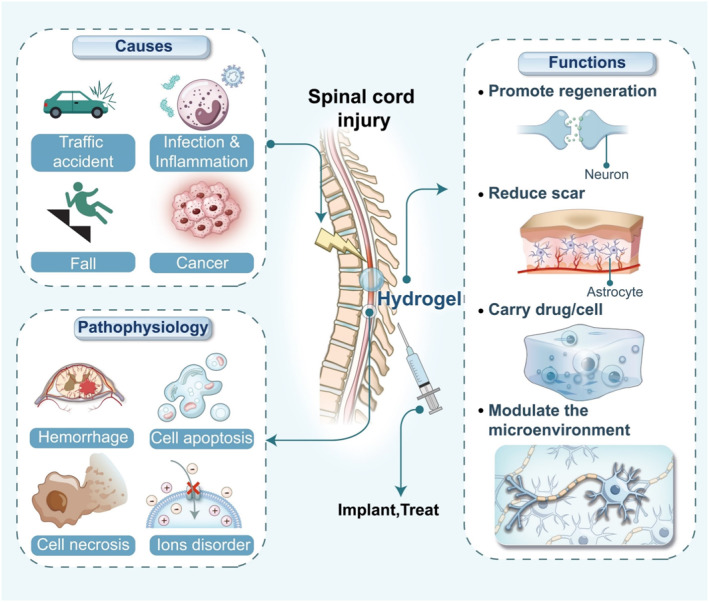

## 1 Introduction

**FIGURE 1:**
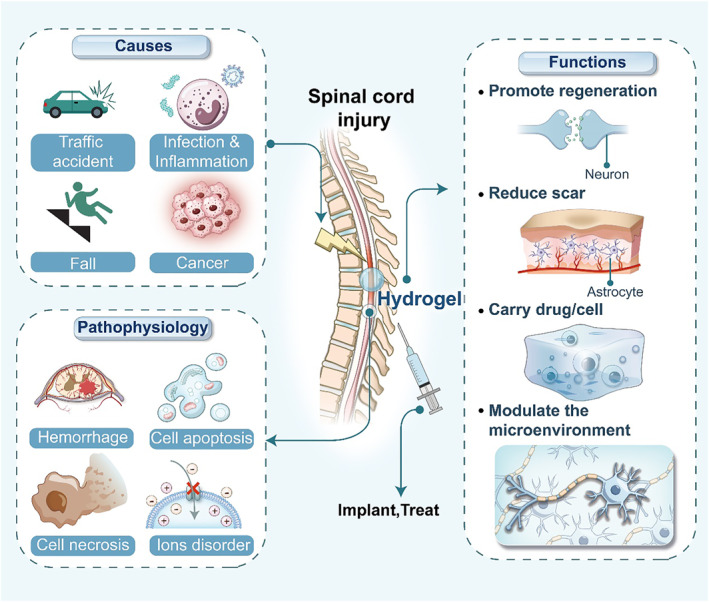
Schematic diagram of causes and pathophysiology of SCI, and functions of hydrogel in SCI treatment.

SCI is divided into traumatic and non‐traumatic types based on the cause of occurrence [1]. The common etiologies of traumatic SCI are traffic accident, fall, sports injury or violence, gunshot wound, etc. The causes of the non‐traumatic SCI are cancer, infection, ischemia, etc. [1, 2] The prevalence and incidence rate of SCI has a substantial increase in the past 30 years on a global scale [3]. Because of the loss of self‐care ability in patients after SCI, the nursing expenses of patients are a huge burden [4]. According to the research data, the lifelong medical expenses caused by SCI may be as high as millions of dollars per person and huge social costs [4, 5]. Current clinical treatments include the use of corticosteroids and mannitol to reduce inflammation and swelling of the spinal cord and laminotomy decompression to relieve local tissue compression [6, 7]. However, these treatment methods cannot have enough effects on the neuronal regeneration and secondary injury (inflammatory response, cell apoptosis, oxidative stress) that play a significant role in the subsequent functional recovery of patients (Figure 1) [7, 8, 9]. Meanwhile, the timing of SCI surgery is still controversial [10, 11].

### 1.1 Mechanisms of SCI

SCI can be divided into two types (traumatic and non‐traumatic) as mentioned before. In this part, we will concentrate on the traumatic SCI.

**FIGURE 2:**
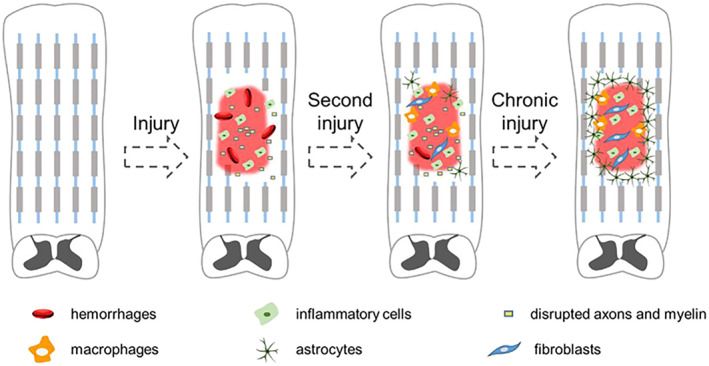
Schematic diagram of SCI. The first injury resulted in direct mechanical destruction and bleeding of the tissue structure. The secondary injury is a complex biochemical cascade reaction caused by the primary injury, which leads to the expansion of the scope of the injury, and the accumulation of inflammatory cells (such as neutrophils, lymphocytes) and macrophages in the injury area. At the stage of chronic injury, the inflammatory response subsided, and the tissue entered repair and scar formation. Reproduced with permission [14]. Copyright 2022, The Authors, published by Elsevier.

In pathophysiology, SCI can be divided into primary and secondary injuries [4, 12]. Primary injuries refer to a series of injury manifestations caused by the high energy accidents (traffic accident, fall, sports injury or violence, gunshot wound, etc.) that impact the spine, such as transient or persistent compression, stretching, laceration, transection. Secondary injuries refer to the further damage caused by biochemical and cellular processes, such as inflammation, ion disorder, apoptosis, and necrosis. With a series of cascade reactions in the process of secondary injuries making further damages to the body, glial scar which will hinder nerves regeneration form at the injured site and finally make a consequence of body dysfunction (Figure 2) [13, 14]. In general, secondary injuries are more complex and serious than primary injuries.

**FIGURE 3:**
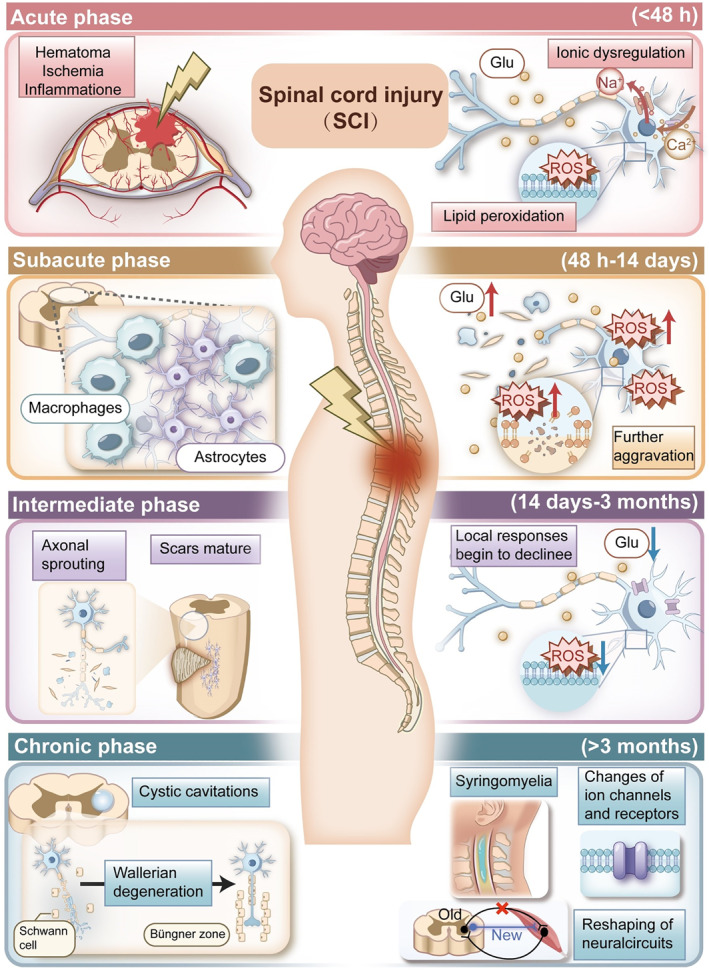
The main pathological changes in each stage of SCI.

According to the time of injury, SCI can be divided into four phases: acute (< 48 h), subacute (48 h–14 days), intermediate (14 days–3 months), and chronic (> 3 months) [4, 15, 16]. We have compiled the main pathological changes in each stage of SCI (Figure 3).

#### 1.1.1 Acute Phase (< 48 h)

At the earliest stage of this phase, high energy events impact the spine and then cause fracture or malposition locally, leading to the compression or damage to the spinal cord. Because of the injured vessels, hematoma forms locally and causes the compression and ischemia of the spinal cord tissue. The increasing cell permeability of injured vessels leads to the local inflammatory response. Meanwhile, there are also some other responses that further exacerbate cell damage, such as ionic dysregulation, cell damage, and lipid peroxidation caused by reactive oxygen species (ROS), calcium dysregulation, and excitotoxicity caused by accumulated glutamate [16, 17, 18, 19].

#### 1.1.2 Subacute Phase (48 h–14 Days)

The characteristics of this phase are immune responses of macrophages and proliferation of astrocytes [8]. In this phase, macrophages infiltrate the injured site and begin to clear the cellular debris. Astrocytes proliferate and further form glial scars that can limit the reaction of inflammatory cells and protect local tissues, but this also has a negative influence on nerve regeneration because glial scars are a barrier that can prevent axon growth [1, 12, 20]. Some responses in the acute phase will further aggravate in this phase, such as free radical injury, lipid peroxidation, and excitotoxicity [12, 20, 21].

#### 1.1.3 Intermediate Phase (14 Days–3 Months)

Astrocytic scars mature continuously and axonal scars start to sprout in this phase [8, 18]. Meanwhile, the condition of SCI tends to stabilize because local responses that can damage cells begin to decline and the glial scars as a barrier protect the cells from the imbalanced microenvironment.

#### 1.1.4 Chronic Phase (> 3 Months)

This phase is characterized by the appearance of cystic cavitations, Wallerian degeneration, which contains axonal degeneration and myelin degeneration, glial scars further maturation [8, 12]. And some other events happen, such as the changes in ion channels and receptors, reshaping of neural circuits, and syringomyelia [22].

### 1.2 Current Clinical Treatments of SCI

At present, the common clinical treatment includes: pre‐hospital emergency care, pharmaceuticals, surgery, physiotherapy, cellular transplantation, and rehabilitation training [6, 23, 24]. In addition to medication and surgical treatment during hospitalization, the treatment before arriving at the hospital and the rehabilitation training after discharge also play an important role in the recovery of patients' physical function.

#### 1.2.1 Pre‐Hospital Emergency Care

SCI is an emergency injury that requires immediate medical intervention in order to stabilize the patient's condition, prevent further injury, and improve the prognosis. At the scene of an injury accident, medical personnel should first evaluate the patient's condition (airway, breathing, circulation, and spine) [4, 6, 25]. Before arriving at the hospital, the following points are needed:

- Monitoring and ensuring the stability of the patient's respiratory tract, respiration and circulation.
- Avoiding moving the injured person unless the patient is in a hazardous environment. At least three people shall use the correct methods such as translation and axial rotation to quickly complete the handling and transfer.
- Fixing the spine with a bracket or spinal fixation plate to prevent additional injury.

#### 1.2.2 Pharmaceuticals

At present, the pharmaceuticals for the treatment of SCI can be roughly divided into neuroprotective and neuroregenerative pharmaceuticals. The main role of neuroprotective pharmaceuticals is to reduce secondary damage after injury and protect nerve cells from further damage, while neuroregenerative pharmaceuticals are committed to promote the regeneration and functional recovery of damaged nerves [26].

##### 1.2.2.1 Neuroprotective Pharmaceuticals

Methylprednisolone is a common neuroprotective drug in clinic and functions by reducing membrane lipid peroxidation [26]. However, the use of methylprednisolone is still controversial. A meta‐analysis of steroid treatment for acute SCI shows that methylprednisolone therapy does not have enough benefits on the patients' short‐term or long‐term recovery, and it can increase the risk of the pneumonia and hyperglycemia [27]. A study showed that the use of methylprednisolone within 8 h of injury can have positive effects on SCI and should not be excluded in treating SCI [28].

Riluzole is a sodium channel blocker that can reduce the release of glutamate that can cause neurotoxicity. Studies have shown that it has a neuroprotective effect on patients with SCI, but further research is still needed before its formal application in clinical practice [28, 29].

Minocycline is an anti‐inflammatory and anti‐oxidant antibiotic which works through regulating MAPK and PI3K/Akt signaling pathways [30]. Minocycline has protective effects on interfering animal SCI models, such as reducing the oxidation reaction to reduce local cellular injury [31]. Phase III clinical trials are currently in progress.

##### 1.2.2.2 Neuroregenerative Pharmaceuticals

GM‐1 ganglioside is a kind of glycolipid molecules that exist in the neuronal membranes [32]. GM‐1 ganglioside can promote axonal regeneration in animal models through regulating the TLR4/NF‐κB inflammatory signaling pathway, but it has not been verified in clinical trials [26, 33, 34].

Granulocyte colony‐stimulating factor (G‐CSF) can promote cell growth through the JAK‐STAT pathway [35]. At present, many studies support G‐CSF as a potential drug for the treatment of SCI [36, 37].

4‐aminopyridine (4‐AP) is a potassium channel blocker that can promote nerve recovery and mitigate demyelination by enhancing signal conduction in injured areas [38, 39]. Although the effects of 4‐AP have been verified on animal models in vitro, further clinical trials are still needed.

#### 1.2.3 Surgery

There are controversies about the timing of surgery [10, 11, 40]. Surgery is still a strategy to relieve local acute compression, reduce complications and improve neurological function. The purposes of spinal surgery can be roughly summarized as decompression and fixation. The commonly used methods are anterior approach, posterior approach and combined anterior and posterior approach [41].

#### 1.2.4 Physiotherapy

Common physiotherapy methods include functional electrical stimulation therapy and hyperbaric oxygen therapy.

Functional electrical stimulation therapy refers to applying electrical stimulation to the paralyzed muscles or nerves that innervate paralyzed muscles and this is conducive to the regeneration of damaged synapses [42, 43, 44].

Hyperbaric oxygen therapy can protect nerves through the following approaches: (a) reducing apoptosis; (b) reducing oxidative stress and lipid peroxidation; (c) reducing inflammation; (d) reducing local edema; (e) promoting vessel formation; and (f) enhancing autophagy [45].

#### 1.2.5 Cellular Transplantation

Cellular transplantation is a method of promoting neural repair and functional recovery by transplanting specific cells, such as mesenchymal stem cells (MSCs), neural stem/progenitor cells (NS/PCs), Schwann cells (SCs), olfactory ensheathing cells (OECs), embryonic stem cells (ESCs), induced pluripotent stem cells (iPSCs) [46, 47, 48].

Although the clinical treatment methods are widely used in SCI, the final effect is still limited. Therefore, a new and reliable treatment strategy is urgently needed. The development of hydrogel tissue engineering brings more new treatment strategies to SCI, and many researchers have successfully developed hydrogels that load therapeutic factors to reduce secondary injury and promote nerve regeneration [49, 50, 51, 52].

## 2 Hydrogels for SCI

Hydrogels are a kind of materials with three‐dimensional network structures and they can absorb and retain a large amount of water (can reach or even exceed 90% of their own weight) [53, 54]. Hydrogels have been widely studied in the field of SCI owing to their good characteristics, such as biocompatibility, plasticity, and organizational similarity [14]. They can simulate the extracellular matrix (ECM) environment in organisms and have adjustable mechanical properties, which make hydrogels become ideal scaffold materials in tissue biology engineering [55, 56, 57].

### 2.1 The Classification of Hydrogels

Depending on the source of the hydrogel, hydrogels can be classified into three types: natural hydrogels, synthetic hydrogels and composite hydrogels [10, 58]. Natural hydrogels are widely used because of their high biocompatibility and rich active sites, but their mechanical properties are poor and it is difficult to bear large mechanical loads. Meanwhile, it is easy to degrade or decompose under the influence of the environment (such as enzyme, temperature, pH) [59, 60]. With uncontrollable performance and limited function, natural hydrogels are difficult to meet the complex application requirements. Synthetic hydrogels have the advantages of high customizability and high stability, but chemical modification is usually required to make them bioactive. At the same time, the residues of synthetic crosslinking agents or initiators may cause cytotoxicity or immune response, and most of them are difficult to biodegrade, or the degradation products may be unfavorable to the physiological environment [61]. Natural and synthetic hydrogels have their own advantages: natural hydrogels are superior in biocompatibility and safety, while synthetic hydrogels are superior in adjustability and mechanical properties. The advantages and disadvantages of the two kinds of hydrogels are almost completely complementary, and each cannot independently meet the increasingly complex needs. In order to make up for the shortage of single material, composite hydrogel came into being. It can not only maintain the biocompatibility and bioactivity of natural materials but also introduce the mechanical properties and stability of synthetic materials.

#### 2.1.1 Natural Hydrogels

Natural hydrogels are substances derived from natural macromolecules, such as polysaccharides extracted from organisms, polypeptides or protein components in animals or plants. Common natural hydrogels are chitosan, hyaluronic acid (HA), alginate, collagen, etc. [44, 61] The advantages of natural hydro gels include biodegradability, low toxicity and low cost, but their stability is poor (easy to degrade) and their mechanical properties are weak [59, 61]. Meanwhile, natural hydrogels may have the risks of causing immune response or carrying pathogens [54, 60, 62].

Chitosan is a natural linear polysaccharide derived from the deacetylation of chitin, which is the second most abundant natural polysaccharide in nature [63]. Because of the characteristics of its cationic surface that can combine with negatively charged bacterial cell membrane and thus has antibacterial effect [64, 65]. Chitosan can only be dissolved in acidic solution because of its rich amino and hydroxyl groups, so it is often necessary to introduce chemical groups (such as carboxyl) to expand its application [66]. Many studies have confirmed that chitosan can be used as a good tissue engineering material for the treatment of SCI. For example, Zhang et al. used chitosan as a scaffold to carry human dental pulp stem cells (DPSCs) and then confirmed that chitosan can promote DPSC differentiation through Wnt/β‐catenin signaling pathway, providing a new strategy for the treatment of SCI which is superior to cell transplantation alone [67]. However, a major limitation of chitosan hydrogels is their relatively low mechanical strength, and their degradation rate can be difficult to precisely control. Moreover, the accumulation of acidic degradation products at the local site may trigger inflammatory responses.

HA is a kind of high molecular polysaccharide formed by disaccharide units (D‐glucuronic acid and N‐acetylglucosamine) linked by β‐1,3 and β‐1,4 glycosidic bonds. HA has strong hydrophilicity and water retention ability owing to its abundant hydroxyl and carboxyl groups. Due to the property that it can combine with water far more than its own weight, HA can form a three‐dimensional network structure filled with water to simulate the ECM environment [56]. The characteristic of HA is its widespread presence in the central nervous system; therefore, using HA as a scaffold to repair SCI has good biocompatibility. However, HA could not form stable hydrogel in its natural form and must be chemically modified, which may partially change its natural properties; at the same time, its degradation rate is too fast to provide long‐term physical support.

**FIGURE 4:**
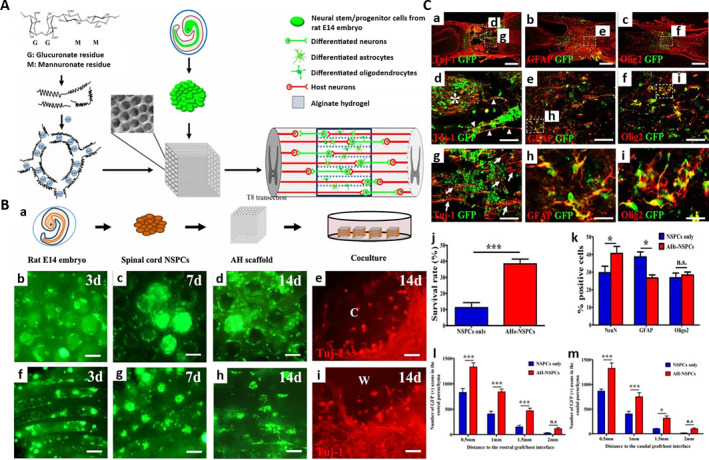
(A) Schematic diagram of synthesis and mechanism of AHs seeded with NS/PCs, and could effectively promote nerve regeneration and synapse formation after SCI. (B): (a) Schematic diagram of NS/PCs implanted into AHs. (b‐i) NS/PCs could survive and proliferate in AHs, and gradually differentiate into neurons over time. (C): (a‐i) Effects of cell differentiation and host axonal regeneration after transplantation of NS/PCs into the transected SCI site without AHs. (j‐m) AHs could provide favorable microenvironment and physical guidance for NS/PCs, improve the survival of transplanted cells and neuronal differentiation, and promote the axon connection. Reproduced under terms of the CC‐BY license [69]. Copyright 2022, The Authors, published by Oxford University Press.

Alginate is a natural linear polysaccharide extracted from brown algae, composed of β‐D‐mannuronic acid and α‐L‐guluronic acid linked by 1,4‐glycosidic bonds. It can quickly cross link with metal ions to form a hydrogel that has three‐dimensional network structures for its molecular structure that contains a large number of carboxyl groups. Alginate hydrogel (AH) shows great potential in the treatment of SCI as a carrier of bioactive molecules [68]. Zhou et al. fabricated alginate hydrogels seeded with NS/PCs and verified that it could increase axonal regeneration (Figure 4) [69]. Grulova et al. developed an alginate scaffold that carried trophic growth factors (GFs) and could continuously release GFs to protect the spinal cord tissue [70]. The disadvantage of alginate is that it lacks cell‐specific adhesion sites, and its degradation depends on non‐specific ion exchange, so it is difficult to achieve active degradation matching with tissue regeneration.

**FIGURE 5:**
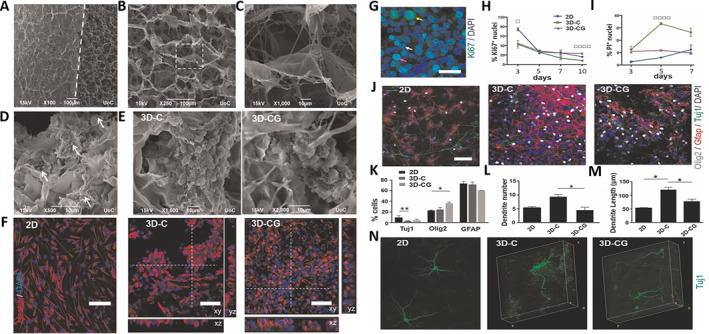
(A‐C) SEM images of the structure of PCSs. (D, E) SEM images of the growth status of NSCs within scaffolds. (F) Confocal fluorescence images of nestin‐positive. (G) Confocal fluorescence image of Ki67‐positive NSCs within the 3D‐CG scaffold. (H) Quantitative analysis of the percentage of Ki67‐positive nuclei. (I) Quantitative analysis of the percentage of PI‐positive nuclei. (J) Confocal fluorescence images showing NSC differentiation in three groups. (K) Quantitative statistics of the proportion of Tuj1‐positive, Olig2‐positive, and GFAP‐positive cells in the three groups. (L‐M) Quantitative analysis of dendrite number and mean dendrite length of NSC‐derived Tuj1‐positive neurons in the three groups. (N) 3D reconstruction of confocal z‐stacks of NSC‐derived Tuj1‐positive neurons. Reproduced under terms of the CC‐BY license [71]. Copyright 2020, The Authors, published by Springer Nature.

Collagen is the most abundant and widely distributed protein in mammals. The structure of collagen is highly similar to the original matrix of tissue, which could provide simulated biochemical signals for cells to support cell adhesion, spreading and proliferation. Alexandra et al. fabricated NSC‐seeded porous collagen‐based scaffolds (PCSs) to promote stable axonal elongation and reduce astrocyte proliferation at the site of SCI (Figure 5) [71]. Fan et al. used collagen scaffolds modified with a collagen‐binding EGFR antibody Fab fragment (CBD‐Fab) to promote neurogenesis of endogenous NSCs activated by injury [72]. However, collagen has a potential immunogenicity risk and high purification cost.

#### 2.1.2 Synthetic Hydrogels

Synthetic hydrogel is a kind of artificially synthesized hydrophilic polymer, which is a three‐dimensional network structure material that can absorb and maintain a large amount of water through chemical or physical cross‐linking. Common synthetic hydrogels are polyethylene glycol (PEG), polyvinyl alcohol (PVA), poly(hydroxyethyl methacrylate) (PHEMA) etc. [53, 56, 61].

PVA is widely used in tissue engineering of SCI due to its excellent biocompatibility and mechanical properties [73]. Its main limitation is that its highly hydrophilic and chemically inert surface leads to poor cell adhesion; at the same time, PVA is difficult to degrade in vivo, and long‐term retention may cause chronic foreign body reaction. Barbon et al. found that PVA could form intelligent scaffolds with biodegradability and protein release ability through halogen mediated partial oxidation, and this modification method not only maintained the molecular weight of PVA but also enhanced its biocompatibility [74]. Liu et al. developed a phase‐separated anisotropic PVA hydrogel loaded with tetramethylpyrazine (TMP/OPH); the hydrogel was fabricated using a combined sol‐gel transition and freeze‐casting method, resulting in an oriented porous structure that mimics the natural extracellular matrix. TMP/OPH demonstrated mechanical properties matching those of native spinal cord tissue and enabled the sustained local release of TMP [75]. In addition, Zhang et al. recently observed and confirmed the piezoelectric properties of PVA for the first time. This discovery makes it possible to use PVA to develop hydrogels that transform the mechanical micro motion in vivo into therapeutic electrical signals, which brings new research directions for the repair of SCI [76].

PEG is a polymer polymerized from ethylene oxide with water or ethylene glycol, which has good water solubility, lubricity, moisturizing properties, and is usually non‐toxic and non‐irritating. But its disadvantages are also obvious: unmodified PEG lacks cell recognition sites, which is not conducive to cell adhesion and growth. PEG has hydrophilicity, solubility, and flexible terminal modification ability, making it easy to covalently bind proteins, peptides, or nanoparticles; the mechanical properties and pore structure of hydrogels can be precisely controlled by adjusting the degree of polymerization, crosslinking density and initiator [77]. Meanwhile, PEG can repair cell membranes and protect mitochondria by reducing secondary damage cascade induced by Ca^2+^, thereby reducing the impact of SCI [78, 79]. Yang et al. used PEG chemical modification to integrate the IKVAV peptide, create a gradient environment and optimize cell behavior [80].

PHEMA has the advantages of excellent biocompatibility and controllable mechanical properties, which makes it one of the ideal scaffold materials in the repair of SCI. Its disadvantage is that the residual monomer may be cytotoxic; at the same time, its polymer network is relatively dense, which may hinder the diffusion of nutrients and metabolic waste. Kubinová et al. modified the SIKVAV (Ser–Ile–Lys–Val–Ala–Val) on PHEMA to promote tissue bridging and axonal directional growth in the cross‐section of the spinal cord; the scaffold not only provided physical support but also promoted nerve regeneration through biochemical signals [81].

#### 2.1.3 Composite Hydrogel

**FIGURE 6:**
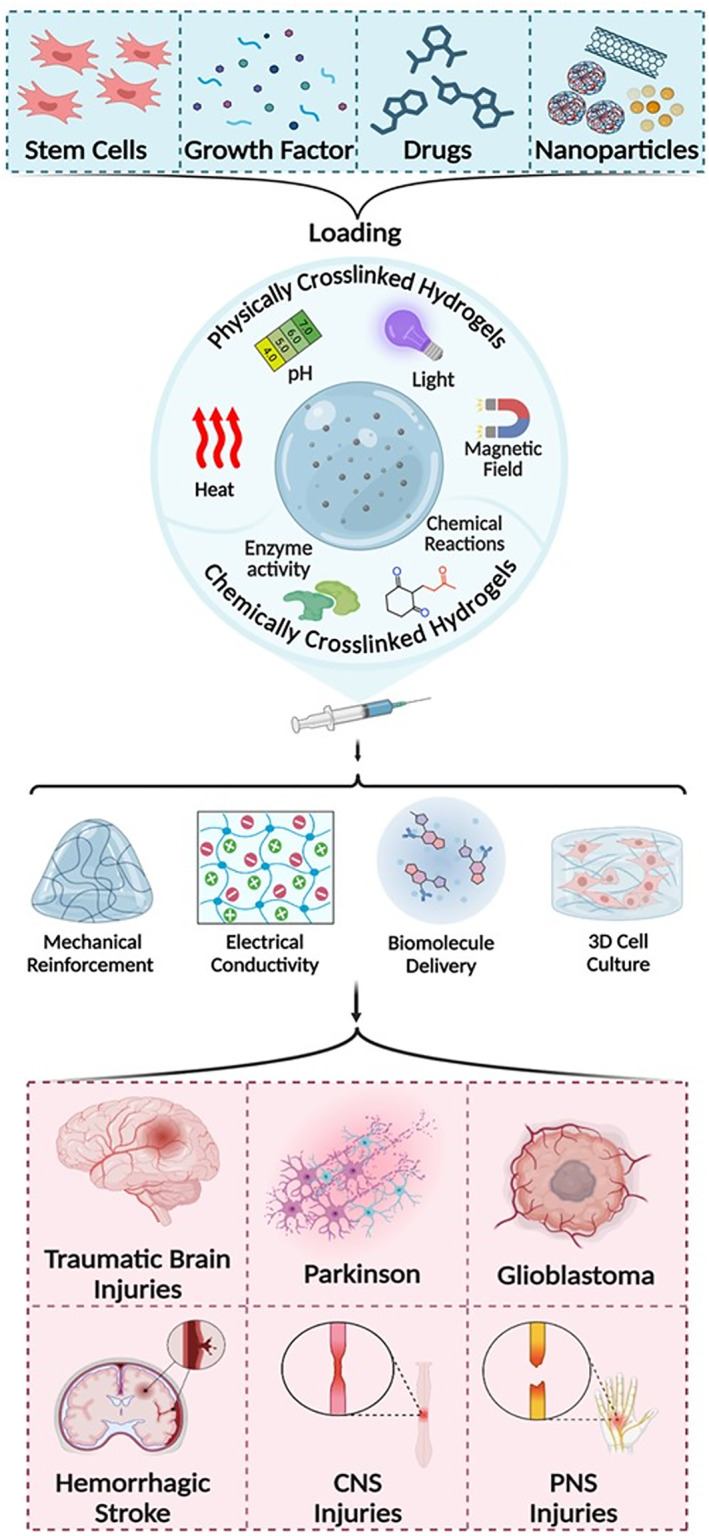
Schematic diagram of the application principle of stimulus‐responsive composite hydrogel in the repair of in nervous system injuries and diseases. Reproduced with permission [83]. Copyright 2025, Elsevier.

The design of composite hydrogel aims to provide a biomaterial, which not only has good biocompatibility but also pursues a balance between mechanical properties, functional activity and environmental responsiveness, which can be regulated and oriented to specific applications. Its core idea is multi‐dimensional design, overcoming the limitations of a single material, and creating a synthetic ECM that can dynamically interact with biological organisms. For example, stimulus responsive composite hydrogels could respond to specific stimuli (such as pH, temperature, light and enzyme activity), so as to achieve dynamic regulation of drug release and tissue regeneration (Figure 6) [82, 83]. Composite hydrogels could precisely regulate drug release, antimicrobial activity and tissue regeneration by integrating a variety of response mechanisms. In addition, the composite hydrogels could also dynamically adjust the mechanical properties of ECM by introducing nano materials, so as to better support the proliferation and differentiation of cells [84, 85].

**FIGURE 7:**
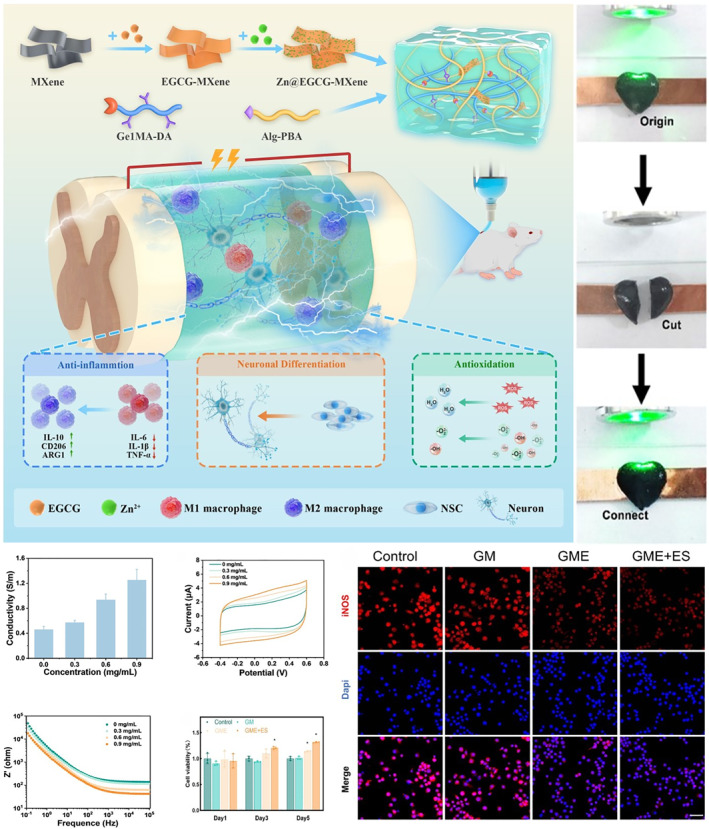
GME hydrogel has high biocompatibility. At the same time, the impedance is low and the conductivity is stable, which is conducive to the rapid propagation of nerve signals. GME hydrogel reduced M1 phenotypic marker (iNOS) and increased M2 phenotypic marker (Arg‐1), indicating that it promoted anti‐inflammatory microenvironment. Reproduced with permission [88]. Copyright 2025, The Authors, published by Springer Nature.

Among the numerous composite hydrogels used in the repair of SCI, conductive hydrogels have attracted much attention due to their unique biological functions. Spinal cord tissue has electrical activity, and nerve signal transduction depends on electrophysiological activities. Appropriate electrical stimulation could effectively accelerate the growth of axons [86, 87]. Conductive hydrogels could partially reconstruct the interrupted electrical signal transmission path after implantation in the injured site, which could effectively promote the repair of the injured nerve. The core value of conductive hydrogels is that they not only act as physical scaffolds to fill the defect but also actively simulate the electrophysiological microenvironment of nerve tissue, and realizes precise intervention on the pathological process of complex injury by integrating a variety of biological functions. For example, Du et al. developed GME hydrogels that were highly conductive via MXene nanosheets, while Zn@EGCG released to achieve ROS clearance and macrophage M2 polarization, so as to synergistically regulate the damage microenvironment and promote nerve regeneration (Figure 7) [88]. He et al. developed an injectable electroactive nano hybrid hydrogel by incorporating raw carbon nanotubes (pCNTs) into functional self‐assembled peptides (SAP), which achieved excellent conductivity and biocompatibility and significantly promoted the recovery of SCI [89].

## 3 Functional Hydrogels in SCI Treatment

Hydrogel has many applications in the repair of SCI. They effectively promote the repair of SCI by providing appropriate mechanical support with a soft nature similar to that of spinal cord tissue, regulating the microenvironment to support the recovery, carrying and releasing drugs or cells that are insufficient at the injury site but needed to repair the local tissue. These characteristics make hydrogel a potential therapeutic strategy in regenerative medicine.

### 3.1 Provide Physical Support Structure

**FIGURE 8:**
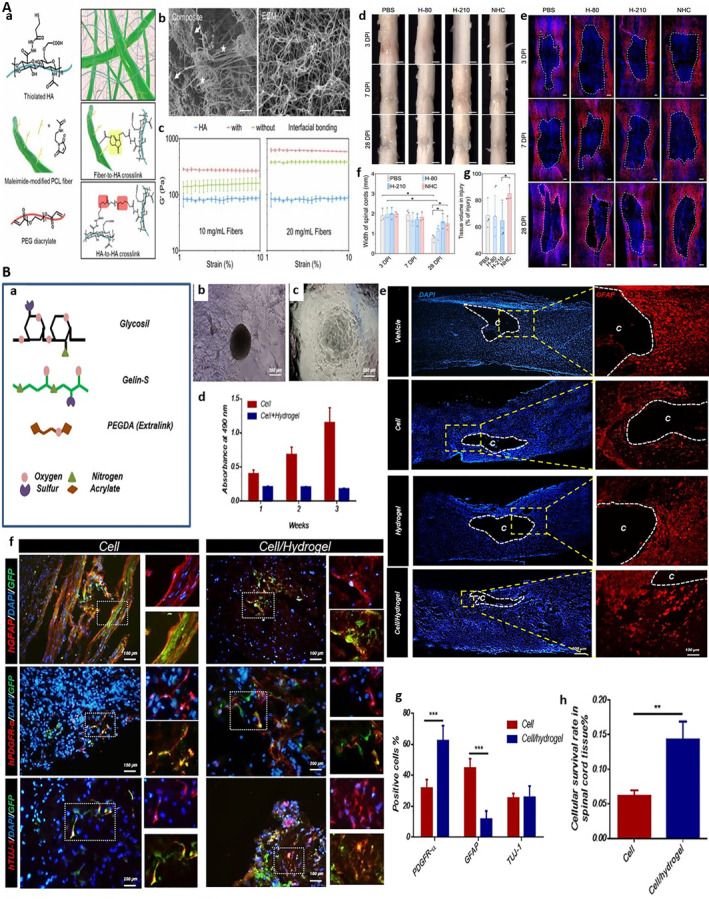
(A): (a‐c) Schematic and structural characterization of the NHC. (d‐g) In vivo anatomical effects of the NHC in a rat spinal cord contusion model. Reproduced with permission [90]. Copyright 2020, Elsevier. (B): (a) Chemical structures of the three core components of the HyStem‐C hydrogel system. (b‐c) Bright‐field images of hESC‐NS cultured on coated plates. (d) MTS assay quantifying the viability of hESC‐NS. (e) Representative immunofluorescence images of longitudinal spinal cord sections. (f) Representative immunofluorescence images showing the in vivo differentiation of GFP‐labeled hESC‐NS. (g) Quantitative analysis of the percentage of PDGFR‐α‐positive, GFAP‐positive, and TUJ‐1‐positive cells among the transplanted hESC‐NS. (h) Quantitative comparison of the survival rate of transplanted hESC‐NS. Reproduced with permission [93]. Copyright 2020, Elsevier.

After SCI, pathological changes such as neuron death, demyelination and glial scar formation make the spinal cord difficult to repair itself. Although cell transplantation (such as NSCs) has broad prospects, it is subject to the severe challenge of low cell survival and retention rate after transplantation. Therefore, hydrogels have become the key to breaking the situation because of their unique three‐dimensional network structure and high water content. On the one hand, hydrogels could provide indispensable physical support for the injured site. Its good mechanical properties help to maintain the structural integrity of the injured cavity, prevent further collapse, and provide a guiding framework for tissue regeneration. On the other hand, as an excellent cell carrier, it could simulate the ECM and provide a protective microenvironment for the adhesion, proliferation and differentiation of transplanted cells, so as to directly solve the problems of low survival rate and poor cell retention rate after cell transplantation. For example, Li et al. developed a nano fiber hydrogel composite (NHC) and transplanted it into the adult rat spinal cord contusion model, and then found it could provide mechanical support for the contused spinal cord, make the tissue width of the injured site twice as large as that of the control group after 28 days of treatment, while promoting the polarization of regenerative macrophages, angiogenesis, axon growth and neurogenesis. Although it was similar to the control group in the volume of spared nerve tissue and hindlimb function, it provided a basis for further optimization of treatment (Figure 8A) [90]. Li et al. prepared a hydrogel scaffold with linear layered structure by coaxial three‐dimensional (3D) printing and the internal double network hydrogel could provide appropriate mechanical support, linear topology and biological activity clues, contributed to the migration and neuronal differentiation of endogenous NSCs, and promoted the recovery of motor function in the rat model of SCI, indicating that the physical support characteristics of hydrogel play an important role in the repair of SCI [91]. In view of the low survival rate of cell transplantation, the three‐dimensional network structure and hydrophilic environment of hydrogel make it an excellent cell carrier, which can significantly improve the retention, survival and function of transplanted cells. Kao et al. developed a system of HA conjugated hydrogel polydopamine nanoparticles (PDA‐NPs) combined with human mesenchymal stem cells (hMSCs). As a multifunctional carrier, the hydrogel had antioxidant and cell delivery functions [92]. Zarei‐Kheirabadi et al. found that wrapping human embryonic stem cell‐derived neural stem cells (hESC‐NS) with HA hydrogel could effectively improve the survival rate and differentiation ability of hESC‐NS, so as to improve the recovery of motor function in rats with SCI (Figure 8B) [93].

### 3.2 Regulate the Local Microenvironment

**FIGURE 9:**
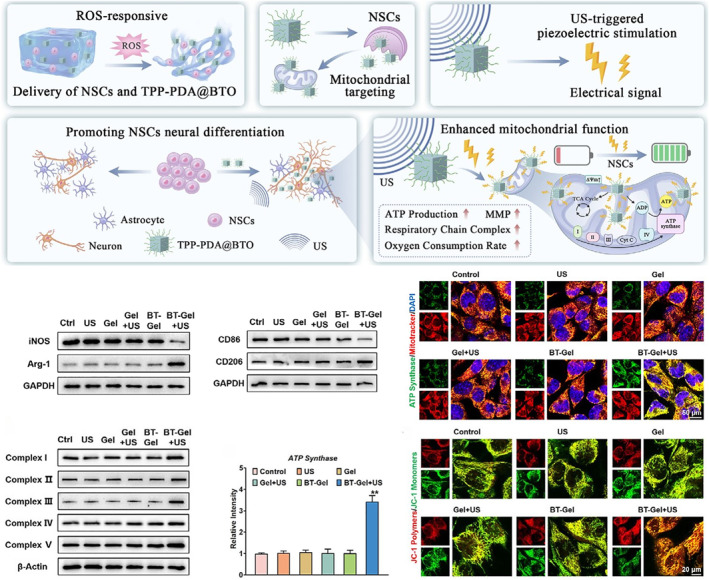
TPP‐PDA@BTO hydrogel targeted mitochondria through piezoelectric stimulation, regulated cell behavior from the metabolic level, and combined immune regulation and intelligent release to optimize the damage microenvironment in multiple dimensions. Reproduced with permission [95]. Copyright 2025, John Wiley and Sons.

Hydrogels can regulate the microenvironment of SCI in a variety of ways, and create favorable conditions for nerve regeneration. For example, Xin et al. formed a hyaluronan‐collagen hydrogel loaded with interleukin‐4 (IL‐4) ZIF‐8 nanoparticles (ZP–DHA–Col) to simulate natural ECM. IL‐4 was released through the response of an acidic microenvironment, which promoted the polarization of macrophages to the M2 type and inhibited inflammation. At the same time, its nano orientation and viscoelasticity enhanced neuron differentiation, axon regeneration, synapse formation and myelin regeneration, and improved the motor function of rats with SCI [94]. Liu et al. developed a type of piezoelectric nanoparticles loaded with mitochondria (TPP‐PDA@BTO) ROS responsive hydrogel (BT‐Gel) that was used to carry NSCs. Under the trigger of ultrasound, hydrogel could regulate the microenvironment by enhancing mitochondrial function and immune regulation (Figure 9) [95].

### 3.3 Drug Delivery Carrier

**FIGURE 10:**
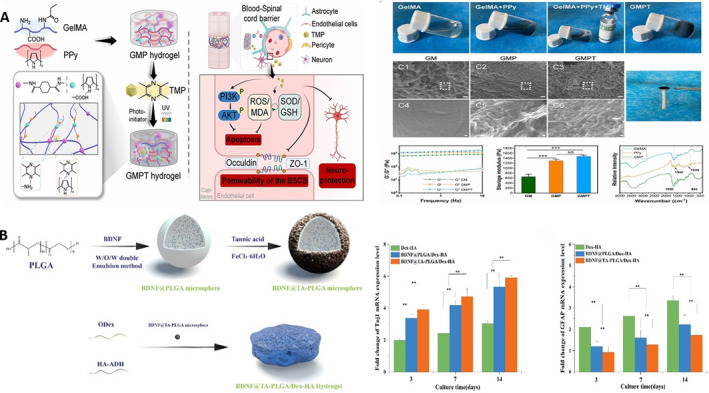
(A) Diagram of the synthesis process, microstructure and mechanical properties of the GMPT hydrogel. Reproduced with permission [97]. Copyright 2024, Royal Society of Chemistry. (B) Diagram of the synthesis process of the BDNF@TA‐PLGA/Dex‐HA hydrogel and its promoting nerve growth effect. Reproduced with permission [98]. Copyright 2021, Elsevier.

Because of the existence of the blood spinal cord barrier (BSCB), the concentration of drugs reaching the SCI site is often insufficient after systemic administration of drugs. BSCB disruption can lead to the invasion of harmful substances (such as inflammatory cells, red blood cells, and complement), exacerbating edema, inflammation, and secondary damage [96]. It seems reasonable that BSCB disruption may allow more drugs to enter, but this chaotic pathological environment, such as increased edema pressure and reduced local blood flow, may actually hinder the effective distribution of drugs to neuronal targets. Therefore, the drug delivery environment in the early stages of injury is extremely harsh. Hydrogel can be used as a drug carrier to realize the controllable release of drugs and improve the curative effect of drugs as a drug carrier. Deng et al. developed the TMP‐loaded conductive hydrogels that could protect BSCB and neurons and promote tissue repair after SCI. The efficacy of TMP as a potential protective agent is often hindered by the limitation of drug delivery route and bioavailability. By combining TMP with conductive hydrogel, the continuous release of TMP at the implantation site could be achieved, which could inhibit oxidative stress response, reduce endothelial cell apoptosis and tight junction protein damage, and finally repair the integrity of BSCB (Figure 10A) [97]. Huang et al. developed a hydrogel composed of oxidized dextran and hyaluronic‐hydrazide as brain‐derived neurotrophic factor (BDNF) delivery systems to promote the differentiation of NSCs into neurons and inhibit the differentiation of astrocytes (Figure 10B) [98]. Wang et al. developed a metal ion assisted self‐assembly complex hydrogel for local delivery of minocycline hydrochloride (MH) that effectively reduced secondary injury and promoted the recovery of motor function in the SCI rat model through local sustained release of MH, which was better than systemic injection of high‐dose MH, indicating that hydrogel could be used as an effective drug delivery system to enhance the inhibitory effect of drugs on inflammatory response [99].

### 3.4 Next‐Generation Intelligent Hydrogels: 4D Printing and Gene Therapy Systems

While conventional hydrogels have shown considerable promise in providing physical support, regulating the microenvironment, and delivering therapeutic agents, their functions are largely pre‐determined and static. Once implanted, they cannot dynamically adapt to the complex, evolving pathophysiological processes of SCI. This limitation has motivated the development of next‐generation intelligent hydrogels that integrate advanced fabrication and genetic engineering to achieve spatiotemporally controlled and adaptive therapeutic interventions.

Four‐dimensional (4D) printing represents an evolution of 3D bioprinting, where the printed constructs are designed to change their shape or function over time in response to specific physiological stimuli [100, 101]. Gene therapy could regulate the expression of specific genes, promote nerve regeneration, inhibit scar formation or regulate inflammatory reaction, and repair the injury from the molecular level. For example, functional units including immunostimulatory sequences, specific aptamers (targeting recognition of cell surface proteins) and responsive sites (such as restriction endonuclease sites) can be designed in hydrogels. This enables a single hydrogel scaffold to integrate multiple functions, such as targeting, drug loading, stimulation response release and immune activation, to achieve collaborative treatment [102].

The convergence of 4D printing and gene therapy makes the potential to create truly intelligent hydrogels. In this system, 4D printing provides a dynamic macro‐architecture that guides tissue organization and responds to the environment, while gene therapy engineers the cellular components at the micro and molecular level to enhance the therapeutic effect. In the future, such a synergistic system could autonomously sense the state of the injury and execute a multi‐stage repair program according to the different phases of SCI.

## 4 Biological Roles of Hydrogels in SCI Treatment

Hydrogel technology for repairing SCI has shown great potential in recent years. The molecular biological effects of hydrogel on spinal cord tissue are mainly reflected in three aspects: local microenvironment regulation, cell behavior regulation and nerve regeneration support. Firstly, hydrogels play an important role in local microenvironment regulation. Hydrogels can reduce secondary injury and the formation of fibrosis scars by regulating the local inflammatory reaction, so as to create more favorable conditions for nerve regeneration. Secondly, hydrogels also show significant advantages in the regulation of cell behavior. Hydrogel can support cell attachment, growth and differentiation through its three‐dimensional structure and biocompatibility. At the same time, due to the regulation of the local damage microenvironment, it also inhibits cell apoptosis. Finally, the application of hydrogels in nerve regeneration support has also made significant progress. Hydrogels can promote axon regeneration and functional recovery by providing physical support and biochemical signals.

**FIGURE 11:**
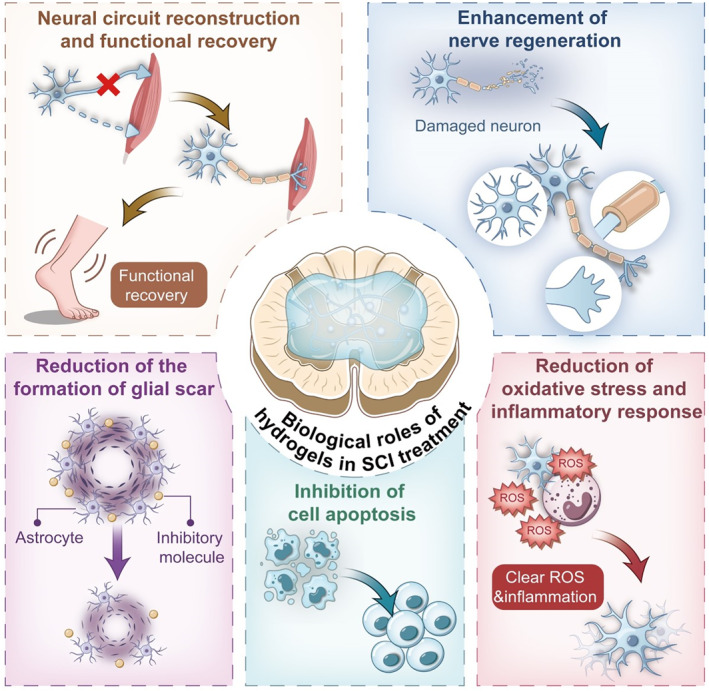
Schematic diagram of biological roles of hydrogels in SCI treatment.

During the phases of SCI, the treatment by hydrogel covers a complete chain from early neuroprotection to late functional recovery. In the acute stage of SCI, hydrogel could achieve immediate protection of nerve tissue by reducing oxidative stress, inhibiting inflammatory reaction and cell apoptosis; In the subacute phase and beyond, it could reduce scar formation by regulating the behavior of glial cells, and provide physical support and biochemical signals for axon regeneration to promote nerve regeneration; Finally, the functional reconstruction of neural circuits is supported by guiding the directional growth of nerve fibers and synaptic remodeling. Hydrogels could dynamically respond to damage microenvironments and implement multi‐stage and integrated repair strategies (Figure 11).

### 4.1 Acute Phase—Reduction of Oxidative Stress and Inflammation

**FIGURE 12:**
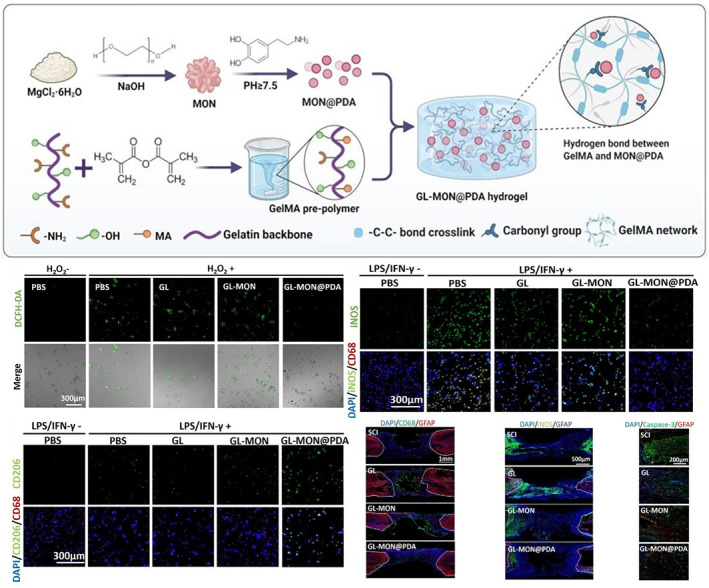
Schematic diagram of the preparation of GL‐MON@PDA mechanism and its ability to resist oxidative stress and neuroprotection. Reproduced with permission [104]. Copyright 2025, Elsevier.

The core pathology of acute and subacute phase of SCI is the outbreak of oxidative stress and inflammatory response. Hydrogel can effectively regulate the local microenvironment after such damage and create a good microenvironment for nerve repair by removing ROS and inhibiting inflammation. For example, Chen et al. designed an injectable ROS and MMP responsive hydrogel (CMV‐RM). Its core lies in the ability to release drugs in a sequential manner according to the pathological microenvironment after SCI: in the early stage of injury, hydrogels respond to high ROS levels and release loaded CNT@MnO_2_ nanomedicine; at the later stage, it releases vascular endothelial growth factor (VEGF) in response to the accumulation of matrix metalloproteinases (MMP) [103]. Wei et al. designed GL‐MON@PDA hydrogel that directly targeted oxidative stress and inflammation, and its PDA shell could rapidly clear ROS, while the release of Mg^2+^ promoted the polarization of macrophages toward M2 type. In vivo experiments confirmed that the hydrogel reduced apoptosis and glial scar formation, highlighting its multi‐target anti‐inflammatory and antioxidant advantages (Figure 12) [104].

### 4.2 Acute and Subacute Phases—Inhibition of Cell Apoptosis

**FIGURE 13:**
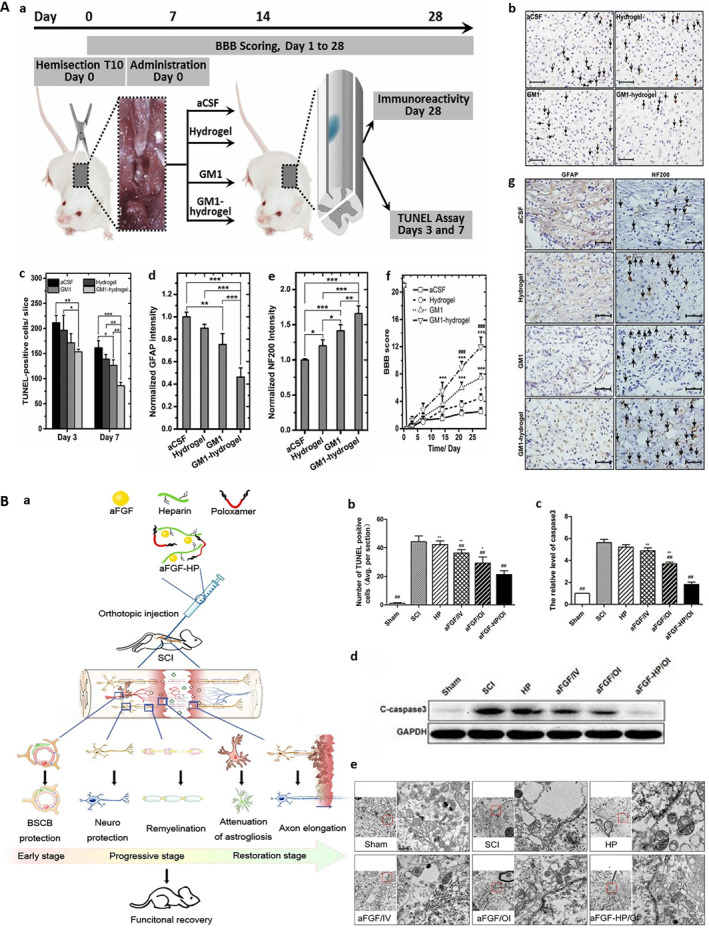
(A): (a) Schematic illustration of the experiment. (b) Representative TUNEL staining images of injured spinal cord sections from each treatment group, with black arrows indicating TUNEL‐positive apoptotic cells. (c) Quantitative analysis of TUNEL‐positive apoptotic cells in the perilesional spinal cord tissue at days 3 and 7 post‐injury for each group. (d) Quantitative analysis of normalized GFAP immunoreactivity in the injured spinal cord at day 28 post‐injury. (e) Quantitative analysis of normalized NF200 immunoreactivity in the injured spinal cord at day 28 post‐injury, indicating the extent of axonal regeneration and neuronal preservation. (f) Longitudinal assessment of hindlimb locomotor function via BBB scores in each treatment group over 28 days post‐SCI. GM1‐hydrogel treatment significantly improved motor recovery from day 14 post‐injury onwards, with sustained superiority up to day 28. (g) Representative immunohistochemical staining images of GFAP and NF200 in injured spinal cord sections from each group at day 28 post‐injury, with black arrows indicating NF200‐positive axons. Reproduced with permission [108]. Copyright 2016, John Wiley and Sons. (B): (a) Schematic of aFGF‐HP hydrogel preparation, in situ administration, and its multi‐stage therapeutic mechanism for SCI repair. (b) Quantification of TUNEL‐positive apoptotic cells in the injured spinal cord. (c) Quantification of relative cleaved caspase‐3 expression. (d) Western blot analysis of cleaved caspase‐3 expression. (e) TEM images of neuronal ultrastructure in the spinal cord of each group. Reproduced with permission [109]. Copyright 2017, American Chemical Society.

Apoptosis after SCI can lead to the death of a large number of neurons and glial cells, which further aggravates neurological dysfunction. The occurrence of apoptosis after SCI is related to the interaction of a variety of signaling pathways, such as P53‐Caspase3 pathway and Fas/Caspase‐8 pathway [105, 106]. In addition, oxidative stress and inflammatory response also aggravate neuronal death by activating apoptosis related signaling pathways, such as the p38/NLRP3 pathway [107]. The activation of these pathways will aggravate the death of neurons. As a new biomaterial, hydrogels have shown their potential in inhibiting cell apoptosis. Liu et al. developed an in situ gelation drug delivery system consisting of a thermosensitive hydrogel matrix based on poloxamer‐407 and 188 mixture and incorporated monosialoganglioside (GM1) and found that it could inhibit apoptotic cell death and glial scar formation, enhance neuronal regeneration, and promote motor function recovery in a rat SCI model by prolonging the release time of GM1 at the injury site (Figure 13A) [108]. Wang et al. formed a thermosensitive heparin poloxamer (aFGF‐HP) hydrogel loaded with basic fibroblast growth factor (aFGF) to protect the bioactivity of aFGF, reduce neuronal apoptosis and reactive astrogliosis, enhance the repair of neurons and axons, and provide an effective method for the treatment of SCI by continuously releasing aFGF (Figure 13B) [109].

### 4.3 Subacute and Intermediate Phases—Reduction of the Formation of Glial Scar

**FIGURE 14:**
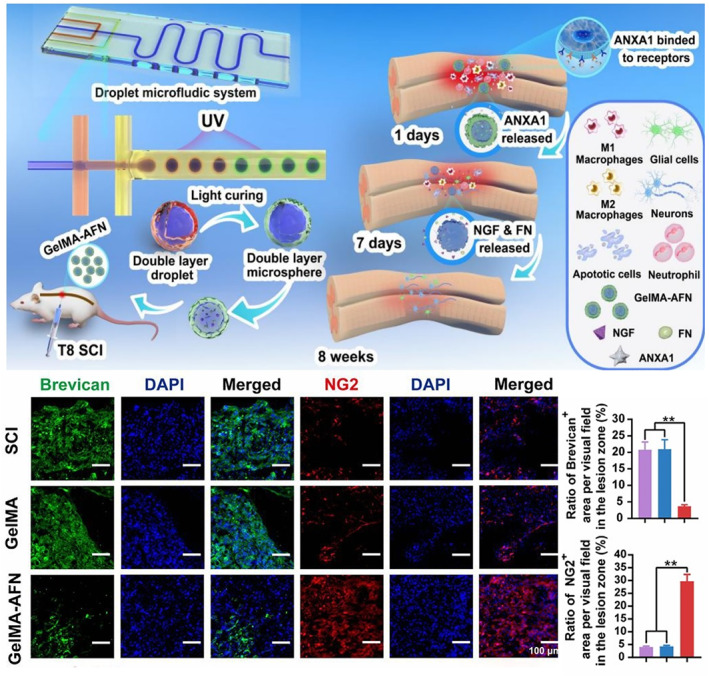
The number of GFAP positive cells in the GelMA‐AFN group was the least, indicating that the glial scar was significantly reduced. Immunofluorescence staining of brevican (scar component) and NG2 (promoting nerve regeneration) showed that the expression of brevican was significantly decreased in the GelMA‐AFN group, while the expression of NG2 was increased, indicating that the hydrogel regulated the scar component to promote regeneration. Reproduced with permission [110]. Copyright 2026, Ivyspring International Publisher.

The core challenges of the subacute and intermediate phases of SCI are the inhibition of glial scar formation, which may lead to the block of the axon regeneration. Glial scar is the main obstacle after SCI and is composed of activated astrocytes and inhibitory molecules (such as CSPG), which hinder axonal regeneration. Hydrogel could reduce the formation of glial scar by following mechanisms: (1) Physically filling the damaged area to reduce cavity formation; (2) Regulating astrocyte activation and CSPG components, and inhibiting inhibitory microenvironment; and (3) Releasing anti‐inflammatory and neurotrophic factors to optimize the repair process. For example, Liu et al. developed GelMA‐AFN microspheres with a core–shell structure. The outer layer of GelMA‐AFN microspheres could release ANXA1 anti‐inflammatory protein, and the inner layer continuously released NGF and fibronectin (FN), realizing stage matching immune regulation and nerve repair. The effect of reducing scar was mainly reflected in inhibiting inflammation in the early stage (reducing neutrophil infiltration and M1 macrophages) and regulating scar components in the late stage (such as reducing brevican expression) (Figure 14) [110]. Yang et al. used GelMA‐F127DA hydrogel as a carrier to wrap OECs for transplantation in the treatment of SCI. The hydrogel provided mechanical support through its 3D porous structure and reduced secondary inflammatory reaction, thus inhibiting glial scar formation and promoting nerve regeneration [111].

### 4.4 Intermediate Phase—Enhancement of Nerve Regeneration

**FIGURE 15:**
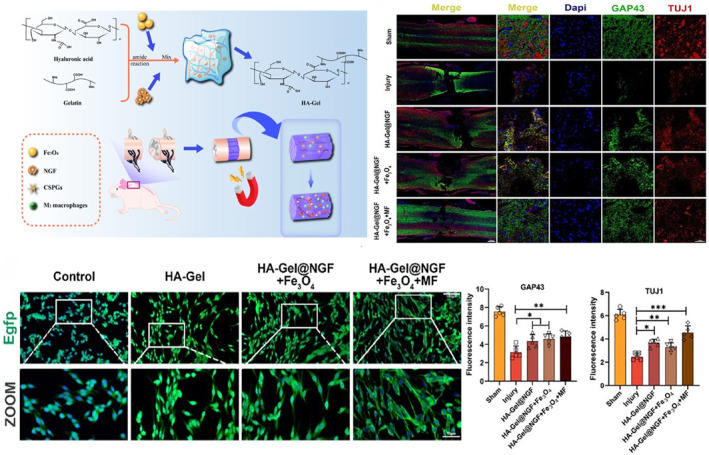
Synthesis process of HA‐Gel@NGF + Fe_3_O_4_ hydrogel and its effect on the repair of SCI. Reproduced with permission [112]. Copyright 2025, The Authors, published by Elsevier.

The core task of the intermediate phase of SCI is to promote axonal regeneration and myelin repair. Hydrogel plays an important role in promoting nerve regeneration at the site of SCI. Studies have shown that a variety of hydrogels provide favorable conditions for nerve regeneration in different ways. Fan et al. developed HA‐Gel@NGF + Fe_3_O_4_ hydrogel loaded with NGF and Fe_3_O_4_ nanoparticles that could respond to hyaluronidase degradation and release neurotrophic factors at the injured site. At the same time, external magnetic field stimulation can improve the microenvironment and promote axon regeneration (Figure 15) [112]. In addition, it is still a major challenge to guide the directional growth of regenerated axons and reconnect them to the correct target, and hydrogel could be a solution strategy. For example, Wen et al. formed a composite scaffold composed of HA hydrogel with longitudinal multi tubular structure and PLGA microspheres loaded with BDNF and VEGF could promote the formation of a large number of neovascularization and regenerated nerve fibers, guide axon growth through the channels, and create a suitable microenvironment for nerve regeneration [113]. Ye et al. developed a GM‐RA4IV hydrogel, which carried a laminin‐derived peptide and was structured with internal parallel microchannels. This design integrated physical topological guidance with biochemical signaling to direct axonal regeneration, resulting in highly ordered parallel growth [114].

### 4.5 Chronic Phase—Neural Circuit Reconstruction and Functional Recovery

**FIGURE 16:**
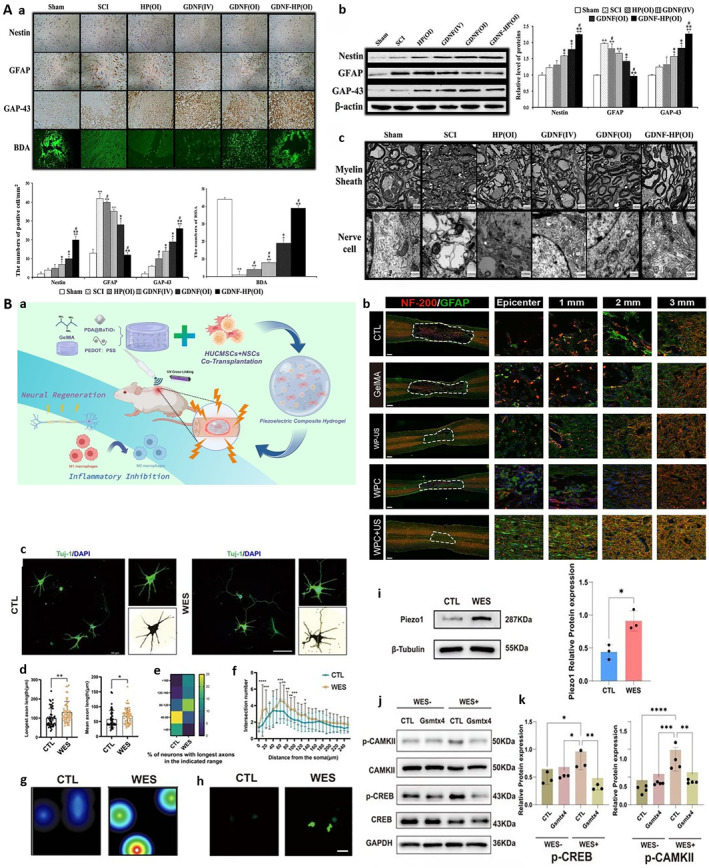
(A): (a) Immunohistochemical staining of Nestin, GFAP, GAP‐43, BDA anterograde tracing images and corresponding quantitative analysis in each experimental group. (b) Western blot analysis and quantification of Nestin, GFAP, and GAP‐43 protein expression in the injured spinal cord. (c) TEM images of myelin sheath and neuronal ultrastructure in the spinal cord of each group. Reproduced with permission [115]. Copyright 2017, John Wiley and Sons. (B): (a) Schematic of the WPC hydrogel system combined with NSCs‐hUCMSCs transplantation for US‐activated wireless electrical stimulation in SCI repair. (b) NF‐200/GFAP double immunofluorescence staining showing axonal regeneration and glial scar formation in the injured spinal cord of each group. (c‐f) Tuj‐1 immunostaining and quantitative analysis of axonal growth and dendritic complexity in primary cortical neurons with or without WES. (g, h) Real‐time calcium imaging and spontaneous firing detection showing enhanced neuronal electrical activity upon WES treatment. (i) Western blot analysis of Piezo1 expression in neurons with or without WES. (j, k) Western blot and quantification of CAMKII/CREB signaling activation in neurons, with Gsmtx4 used to block Piezo1 channel. Reproduced under terms of the CC‐BY license [116]. Copyright 2025, The Authors, published by Elsevier.

The core challenges of the chronic phase of SCI are neural circuit reconstruction and functional recovery. Hydrogels work by providing physical guidance and neurotrophic signals. Studies have shown that the transplantation of nanostructured composite scaffolds can promote the regeneration of nerve fibers and significantly improve motor function in chronic SCI. In addition, hydrogels can also provide continuous neurotrophic support and anti‐inflammatory effects through a responsive drug delivery system, so as to promote the recovery of structure and function. The physical properties of the hydrogel enable it to provide structural support in the damaged area and promote the growth and connection of neurons. For example, Zhao et al. formed a temperature sensitive heparin poloxamer hydrogel to support the role of nerve growth factor (GDNF), promote neural circuit remodeling and neuroprotection, and improve functional recovery after SCI (Figure 16A) [115]. Hydrogel could also be used as a carrier of neurotrophic factors to enhance the effect of nerve regeneration. Zhong et al. developed a wireless piezoelectric hydrogel (WPC) driven by ultrasound and transplanted it with NSCs and umbilical cord mesenchymal stem cells (hUCMSCs); the WPC hydrogel improved the recovery of structure and function after SCI by regulating the immune microenvironment and promoting nerve regeneration (Figure 16B) [116].

In order to better show the advances in hydrogel tissue engineering for SCI, we sorted out the hydrogels that appeared in this review (Table 1).

**TABLE 1:** Summary of the hydrogels that appeared in this review.

Name of the hydrogel	Fabrication strategies	Classification	Biological function	Reference
Chitosan porous scaffold	Lyophilization	Natural	Supports cell attachment and neural differentiation, regulates microenvironment, inhibits cell apoptosis and promotes functional recovery	[67]
Alginate hydrogel	Cross‐linked by Ca^2+^	Natural	Promotes the proliferation and differentiation of NSCs, axonal regeneration, and functional recovery	[69]
Alginate scaffold	Cross‐linked by Ca^2+^	Natural	Promotes motor neuron survival, axon regeneration, improves functional recovery and sensory function	[70]
Porous collagen‐based scaffold	Dehydro‐thermal treatment	Natural	Promotes NSCs differentiation, axon elongation and synapse formation, inhibits scar formation, and improves functional recovery	[71]
Modified collagen scaffold	Biological physical crosslinking (protein‐protein interaction)	Natural	Promotes nerve regeneration and differentiation, synapse formation, inhibits scar, regulates microenvironment, and promotes functional recovery	[72]
PVA hydrogels oxidized by halogens	Physical crosslinking (freeze‐thaw cycles)	Synthetic	Adjustable biodegradation potential, mechanical properties matching different biological tissues, potential drug/protein delivery carriers	[74]
TMP/OPH	Physical crosslinking (micro‐crystallization domains induced by phase separation)	Synthetic	Reduces inflammatory response and oxidative stress, provides physical guidance and support, promotes nerve regeneration and reduces scar	[75]
PEGDM	Photopolymerization crosslinking	Synthetic	Provides adhesion microenvironment for stem cell transplantation, promotes nerve regeneration and inhibits cell apoptosis	[80]
SIKVAV‐modified PHEMA hydrogel	Crosslinked by ethylene dimethacrylate (EDMA)	Synthetic	Promotes cell adhesion and growth, guides axon regeneration, tissue integration and bridging, immune regulation	[81]
GME hydrogel	Dynamic covalent crosslinking (borate ester bond) + photopolymerization covalent crosslinking (UV curable GelMA‐DA)	Composite	Conducting electricity, regulates inflammatory microenvironment, promotes nerve regeneration, reduces scar and promotes angiogenesis	[88]
HGF/pCNTs hydrogel	Non covalent interactions (mainly π‐π stacking and cation‐π complementary interactions)	Composite	Promotes the growth of axons, the migration of SCs and the interaction with axons, and enhances the myelination of axons	[89]
NHC	Covalent crosslinking (thiol acrylate click chemical reaction) and interface bonding (chemical bond between fiber and hydrogel network)	Composite	Provides mechanical support to reduce collapse, promotes macrophage polarization to M2 phenotype, enhances angiogenesis, promotes axon growth, supports the survival of neural precursor cells and neurogenesis	[90]
Coaxial 3D printed hierarchical structured hydrogel scaffold	Outer layer: Temperature sensitive physical crosslinking, Inner layer: Covalent crosslinking (Michael addition) and ionic crosslinking	Composite	The outer layer rapidly down to regulate oxidative stress, and the inner layer provides long‐term mechanical support, promotes NSCs migration and neuronal differentiation, reduces scar and promotes functional recovery	[91]
H‐P‐M hydrogel	Chemical crosslinking and physical embedding	Composite	Antioxidant and anti‐inflammatory, tissue adhesion, cell protection and scaffold effect, promotes endogenous nerve regeneration, axon regeneration and functional recovery, immune regulation	[92]
HyStem‐C cell culture scaffold	Thiol ene click chemical crosslinking	Composite	Cell encapsulation and delivery, promotes directional differentiation of neural stem cells, inhibits glial scar, improves motor function, microenvironment simulation	[93]
ZP‐DHA‐Col	Dynamic imine bond crosslinking, photoinitiated covalent crosslinking	Composite	Neuroprotection, nerve inducing, promotes the recovery of functional recovery	[94]
PP‐PDA@BTO	Photoinitiated covalent crosslinking and dynamic phenylborate ester bond crosslinking	Composite	Promotes the differentiation of neural stem cells into neurons, regulates the immune microenvironment, supports nerve tissue regeneration, and improves the recovery of motor function	[95]
GM‐PPy‐TMP hydrogel	Photoinitiated covalent crosslinking	Composite	Protects the integrity of BSCB, inhibits endothelial cell apoptosis, reduces oxidative stress, protects neurons and axons, and promotes the recovery of motor function	[97]
BDNF@TA‐PLGA/Dex‐HA hydrogel	Schiff reaction, supramolecular interactions such as hydrogen bonds and electrostatic interactions	Composite	Conductivity, promotes differentiation of neural stem cells into neurons, controllable degradation, slow release of BDNF, inhibits scar	[98]
Agarose hydrogel loaded with DS‐MH complexes	Physical embedding, metal ion assisted self‐assembly and electrostatic interaction	Composite	Local release of MH, neuroprotection, immune regulation, and improvement of functional recovery	[99]
CMV‐RM hydrogel	Borate ester bond crosslinking, hydrogen bond, doping enhancement (interaction between CNT and MnO_2_)	Composite	Sequential response (in the early stage responses to high ROS and reduce oxidative stress and inflammation, in the late stage responses to MMP to promote angiogenesis and neural stem cell differentiation), inhibits scar and promotes functional recovery	[103]
GL‐MON@PDA	Photoinitiated radical polymerization crosslinking	Composite	Sequential elimination of ROS and continuous release of Mg^2+^, inhibition of calcium overload and excitotoxicity, regulation of immune microenvironment, promotion of nerve regeneration, promotion of angiogenesis, inhibition of scar, promotion of structural repair, and promotion of functional recovery	[104]
GM1 hydrogel	Physical crosslinking (thermosensitive gelation)	Composite	Sustained drug release, neuroprotection and regeneration, inhibits scar, promotes motor function recovery, local formation of barrier (reduce the loss of drugs washed by cerebrospinal fluid)	[108]
aFGF‐HP hydrogel	Chemical crosslinking (EDC/NHS covalent coupling) and physical crosslinking (thermosensitive gelation)	Composite	Sustained release of aFGF, protection of BSCB, neuroprotection and regeneration, inhibition of glial scar, promotion of motor function recovery, inhibition of endoplasmic reticulum stress	[109]
GelMA‐AFN microspheres	Photopolymerization crosslinking	Composite	Spatiotemporal controlled release drugs (the outer layer inhibits inflammation in the early stage, and the inner layer continuously promotes nerve repair), immune regulation, neuroprotection and regeneration, reduces glial scar formation and promotes functional recovery	[110]
GelMA‐F127DA hydrogel	Photopolymerization crosslinking	Composite	Supports the survival and proliferation of OECs, reduces scars, promotes nerve regeneration and functional recovery	[111]
HA‐Gel@NGF + Fe3O4 hydrogel	Amide reaction of amino and carboxyl groups	Composite	Drug delivery and sustained release, promotes nerve regeneration	[112]
HA scaffold containing PLGA microspheres	Amide reaction of amino and carboxyl groups	Composite	Guides the directional growth of axons, regulates the inhibitory microenvironment, promotes angiogenesis, inhibits glial scar, promotes nerve regeneration and integration, and restores motor function	[113]
GM‐RA4IV hydrogel	Chemical crosslinking (peptide bond connection) and photopolymerization crosslinking	Composite	Promotes axonal regeneration and directional growth, inhibits inflammation, reduces glial scar and restores motor function	[114]
GDNF‐HP hydrogel	Physical crosslinking (thermosensitive gelation)	Composite	Promotes nerve regeneration and protection, bidirectional regulation of autophagy (promotion of autophagy dependent cell survival in the early stage, inhibition of autophagy induced apoptosis in the late stage), inhibition of cell apoptosis, and improvement of motor function	[115]
WPC hydrogel	Photopolymerization crosslinking	Composite	The scaffold of transplanted cells, provides in situ electrical stimulation (driven by ultrasound), regulates the immune microenvironment, reduces scar hardness, reduces cyst formation, promotes axonal regeneration and neural function recovery, activates Piezo1 channel and downstream CaMKII/CREB signaling pathway, and enhances neuronal plasticity	[116]

## 5 Conclusion and Perspective

As an advanced material with excellent biocompatibility, adjustable physical and chemical properties and a three‐dimensional bionic microstructure, hydrogels show great potential in the field of SCI repair. This paper reviews the molecular biological functions of a hydrogel in SCI, and its application in the repair of SCI by providing a physical support structure, regulating the microenvironment, and drug delivery carriers. The current research has developed from a simple filling scaffold to a functional and intelligent composite system, which realizes the dynamic intervention of the damage microenvironment. Although hydrogels exhibit considerable promise in drug delivery and tissue engineering, their path to clinical application is constrained by a number of persistent issues. First, achieving precise spatiotemporal control within the complex human physiological environment, balancing material degradation with the pace of tissue regeneration, and reliably translating laboratory efficacy into long‐term functional recovery in patients remain fundamental hurdles. Furthermore, the very tunability of hydrogels that enables diverse biomedical functions introduces complications in reproducible manufacturing and scalable production. Variations in complex chemical compositions can lead to batch‐to‐batch inconsistencies, undermining clinical feasibility. Additionally, many hydrogel systems and incorporated bioactive factors are incompatible with conventional high‐temperature or high‐pressure sterilization methods, posing a significant barrier to clinical adoption and patient safety.

In the future, the design and preparation of hydrogels will pay more attention to simulating the structure and function of natural ECM, so as to better promote nerve regeneration. For example, by accurately regulating the chemical composition, microstructure and mechanical properties of hydrogel, it can more accurately simulate the microenvironment of spinal cord tissue, providing more suitable conditions for the survival, proliferation and differentiation of nerve cells [117, 118]. In addition, the realization of “spatiotemporal controlled release” of therapeutic molecules will be another research focus. In addition, the realization of the “spatiotemporal controlled release” of therapeutic molecules is another research focus. For example, in peripheral nerve regeneration, Yao et al. matched the release curve of Mg^2+^ to the critical period of SC proliferation, migration and myelination through a multi ‐ gradient design (using magnesium compounds with different degradation rates in the inner and outer layers) [119]. This provides a new idea for the design of intelligent hydrogels for SCI, which could sequentially release anti‐inflammatory factors, neurotrophic factors.

The application of 3D printing and 4D printing technology will bring new breakthroughs in the preparation of hydrogels. These technologies can realize the customized manufacture of hydrogels. According to the specific situation of SCI, hydrogel scaffolds with specific shape, structure and function can be accurately designed and manufactured to improve the pertinence and effectiveness of treatment [118]. At the same time, the development of intelligent hydrogels is also an important direction. This kind of hydrogel can respond to environmental stimuli such as temperature, pH, electric field, magnetic field, etc., realize the on‐demand release of drugs and the dynamic regulation of cell behavior, and further improve the therapeutic effect [120]. In addition, the combination of hydrogel and other advanced technologies, such as gene therapy and nanotechnology, is expected to develop more innovative treatment strategies.

## Author Contributions

**Ruixing Shui:** conceptualization, writing – original draft, investigation. **Fan Ding:** assistant for writing – original draft, validation. **Dapeng Li:** conceptualization, writing – review and editing, supervision, funding acquisition. **Guoqing Pan:** conceptualization, writing – review and editing, supervision, funding acquisition. All authors have read and agreed to the published version of the manuscript.

## Ethics Statement

The authors have nothing to report.

## Conflicts of Interest

The authors declare no conflicts of interest.

## Data Availability

The data that support the findings of this study are available on request from the corresponding author. The data are not publicly available due to privacy or ethical restrictions.
